# Erectile Dysfunction Under Age 40: Etiology and Role of Contributing Factors

**DOI:** 10.1100/tsw.2004.64

**Published:** 2004-06-07

**Authors:** Tahir Karadeniz, Medih Topsakal, Ahmet Aydogmus, Dogan Basak

**Affiliations:** Okmeydani Training Hospital, Istanbul, Turkey

## Abstract

The aim of this study was to evaluate the etiology of erectile dysfunction (ED) in patients under the age of 40 years. Eighty one patients were included in this study. All patients underwent a multidisciplinary diagnostic approach by color Doppler ultrasonography, dynamic pharmacocavernosometry (optional), selective pudendal pharmaco-arteriography (optional) and nocturnal penile tumescence monitoring by a Rigi-Scan (optional). Mean age of the population was 32 years. Psychogenic impotence was diagnosed in 50% of the patients and organic impotence was diagnosed in 45%. After the 3rd decade of life, a vasculogenic etiology was the most common cause of impotence. Smoking and hypertension played a major role as chronic contributing factors in the overall study population. Primary impotence was diagnosed in 11 patients who were unmarried. The rate of organic causes was 45% in this group (all vasculogenic in nature). Erectile dysfunction in younger patients and in patients with primary impotence is due mainly to organic causes, usually vascular in origin.

## INTRODUCTION

Erectile dysfunction (ED), known as an inability to achieve or maintain erection with sufficient rigidity and duration to permit satisfactory sexual performance, is an age dependent disorder. The prevalence of complete ED in healthy men triples from 5% at 40 years to 15% at 70. ED is more prevalent in patients with other associated medical conditions such as diabetes mellitus, heart disease, hypertension and cigarette smoking.[1] However, sufficient data does not exist in the literature regarding the incidence, etiology and contributing factors leading to ED in the early decades of life. The aim of this study was to evaluate the etiologic factors and the role of associated medical disease in patients up to 40 years of age with ED.

## METHODS

A total of 81 patients participated in this study. All patients were examined with a multidiciplinary approach. The first step of the diagnostic work-up was to obtain a detailed sexual and medical history by a comprehensive interview. The aim of the medical history was to determine the presence of any factors which may be related to ED, such as medications (i.e. antihypertensive agents), heavy smoking (> 10 cigarettes / day), coronary and cerebrovascular disease, diabetes, hypertension, previous pelvic, perineal or penile trauma, prior surgery, history of multiple sclerosis, brain or spinal cord injury, and presence of urinary incontinence. Physical examination consisted of penile palpation for Peyronie's disease, assessment of penile and perianal sensation, anal sphincter tone and response of the bulbocavernous reflex. Serum levels of glucose, cholesterol, prolactin and free testosterone were obtained.

All patients underwent two penile pharmaco-color Doppler sonography examinations. Patients were examined both before and after intracavernous injection of either 60 mg papaverine (57 pts.) or 10 ug prostaglandin E1 (24 pts.). Scanning of cavernosal artery peak systolic flow was performed beginning 5 min. after vasoactive drug injection and repeated at 5 min. intervals up to 20 minutes. Arterial inflow was considered sufficient if greater than 30 cm/sec. If after the end of this period, sufficient erectile rigidity was not achieved, patients were instructed to perform genital manipulation in a private setting. Subjects with sufficient arterial inflow and without sufficient rigidity underwent dynamic pharmaco-cavernosometry using 10 ug. of prostaglandin E1. The results were considered normal if the maintenance flow rate was less than 15 ml/min. and the intracavernosal pressure was more than 60 mm Hg, 5 minutes after the infusion was stopped.[2] Pharmaco-selective pudendal angiography was performed in 4 patients with known pelvic trauma associated with urethral disruption. Thirteen patients had nocturnal penile tumescence monitoring performed by a Rigi Scan. Diagnostic criteria for a normal study was more than 2 erectile episodes per night, a tip rigidity greater than 60%, and a duration of at least 5 minutes.[3]

## RESULTS

The mean age of the patient population was 32 years with a range between 19 and 40. The duration of dysfunction ranged from 3 to 120 months with a mean 19 months. The patients were distributed into 4 age groups. In table 1 the etiologic factors of ED for different age groups are demonstrated. Overall, nicotine abuse and antihypertensive medication were the most common contributing factors to ED. In table 2, the contributing factors to ED and their pathophysiological mechanisms are shown. Eleven of 81 patients were diagnosed with "primary impotence". Their mean age was 30 years. In 6 (54 %), the cause for ED was identified as psychogenic and in 5 the cause was organic.

**TABLE 1**
**Age Distribution of the Patients with Regard to Etiology**

| | Age Group in Years | | | | |
|---|---|---|---|---|---|
| ***Etiology:*** | **19-25** | **26-30** | **31-35** | **36-40** | **Total** |
| *Arteriogenic* | 3 | - | - | 10 | **13** |
| *VOD* | - | 6 | 6 | 2 | **14** |
| *Arteriogenic + VOD* | - | 1 | 1 | 7 | **9** |
| *Psychogenic* | 3 | 14 | 11 | 13 | **41** |
| *Neurogenic* | 2 | - | 1 | 1 | **4** |
| **Total** | **8** | **21** | **19** | **33** | **81** |

**TABLE 2**
**List of Contributing Factors and their Mechanism to Cause ED**

| *Contributing Factors:* | No. of Patients | Pathophysiology |
|---|---|---|
| *Smoking* | 16 | Vasculogenic |
| *History of hypertension or antihypertensive medication* | 3 | VOD |
| | 5 | Pure arteriogenic |
| | 1 | VOD + arteriogenic |
| *Pelvic trauma with urethral dysfunction* | 2 | Pure arteriogenic |
| | 2 | VOD + arteriogenic |
| *DM* | 3 | Vascular |
| | 1 | Neurogenic |
| *Brain Surgery* | 2 | Neurogenic |
| *Radical cysto-prostatectomy* | 2 | Neurogenic |
| **Total** | **37** | |

Pharmaco-angiography revealed an absence of cavernosal artery filling in 4 of the patients with pelvic trauma. In 13 patients, the diagnosis of "psychogenic impotence" was confirmed by color Doppler sonography and/or dynamic cavernosometry, in addition to a Rigi-Scan.

## DISCUSSION

The ratio of organic to psychogenic male sexual dysfunction has been reported to be directly proportional to age; with 70 % of men under 35 years of age having psychogenic ED and 85 % of men over 50 years of age having organic ED.[4] Our results confirm these figures. The rate of psychogenic impotence in the population under 35 years was 58% (28/48). 10 of the 20 patients under 35 years with organic ED had obvious contributing factors such as brain surgery (2), pelvic trauma and urethral disruption (4), radical pelvic surgery (1), and insulin dependent diabetes (3).

The Massachusetts male aging study assessed the prevalence of impotence among 1290 men, ranging between 41 to 70 years, and reached the conclusion that the incidence of ED increased with age and vascular risk factors.[5] In Donatucci and Lue's study the age range of subjects was between 18-40 years and 67% of them were between the ages of 31-40 years. Vasculogenic factors were the leading cause of ED in that study.[6] Davidson et al examined 250 men and found no age related increase in sexual dysfunction before the sixth decade of life.[7] Our investigation revealed that apart from the patients with obvious contributing factors (pelvic trauma, radical cystectomy, brain surgery, diabetes) the majority of the remaining subjects had a vascular cause for ED which occurred between the 3rd and 4th decades of life.

It is well known that ED is seen in patients with manifestations of arteriosclerotic disease and may be a symptom of a systemic vascular problem related to well known risk factors such as smoking, hypertension, hyperlipidemia and DM.[8] Abnormal penile brachial indexes in subjects without a history of stroke or myocardial infarction correlate with ischemic heart or cerebrovascular disease.[9] Besides these factors leading to vasculogenic impotence, antihypertensive medications also interfere with the neurophysiology of erection.[10] All antihypertensive agents, regardless of composition, have been implicated in causing ED.[11] In a study of Muller et al., the incidence of hypertension in patients with ED was 24.8 %.[12] A history of hypertension was present in 13 of our patients over 35 years (see table 2).

Nicotine abuse was also found to be an associated factor leading to ED in our series. We considered more than 10 cigarettes per day as heavy smoking, but it is known that even 2 cigarettes per day may have deleterious effect on erection.[13]

The cause of impotence in unmarried patients is believed to be mainly psychogenic.[14] In our study, 11 of 81 subjects were unmarried and were diagnosed as having impotence. The definition of primary impotence is the inability to take part in intercourse because of psychiatric reasons and/or the absence of full sustained erections due to urological causes since puberty.[15] Lee et al examined 15 patients with primary ED and compared them to 145 psychogenic patients.[14] As in our series, the patients with primary impotence had a history of unsuccessful intercourse and/or erection since puberty, despite the absence of any trauma or definite associated medical disease. Their results revealed mostly a vasculogenic pathology of ED.[14] Similarly, Stief et al. evaluated 67 patients with primary impotence with an age range 18 to 60 years and demonstrated that 85% had an organic cause for their ED.[15]

In conclusion, in young patients without obvious contributing factors, impotence is mostly due to a psychogenic etiology. However, in patients after the 3rd decade of life, a vasculogenic etiology is likely. In patients with primary impotence, the role of an organic etiology should also be considered during the diagnostic work-up.
